# Effects of Hormonal Replacement Therapy and Mindfulness-Based Stress Reduction on Climacteric Symptoms Following Risk-Reducing Salpingo-Oophorectomy

**DOI:** 10.3390/healthcare12161612

**Published:** 2024-08-13

**Authors:** Amira Mohammed Ali, Saeed A. Al-Dossary, Carlos Laranjeira, Faten Amer, Souheil Hallit, Abdulmajeed A. Alkhamees, Aljawharah Fahad Aljubilah, Musheer A. Aljaberi, Ebtesam Abdullah Alzeiby, Hammad Ali Fadlalmola, Annamaria Pakai, Haitham Khatatbeh

**Affiliations:** 1 Department of Psychiatric Nursing and Mental Health, Faculty of Nursing, Alexandria University, Smouha, Alexandria 21527, Egypt; amira.mohali@alexu.edu.eg; 2 Department of Psychology, College of Education, University of Ha’il, Ha’il 55476, Saudi Arabia; s.aldossary@uoh.edu.sa; 3 School of Health Sciences, Polytechnic University of Leiria, Campus 2, Morro do Lena, Alto do Vieiro, Apartado 4137, 2411-901 Leiria, Portugal; 4 Centre for Innovative Care and Health Technology (ciTechCare), Polytechnic University of Leiria, Campus 5, Rua das Olhalvas, 2414-016 Leiria, Portugal; 5 Comprehensive Health Research Centre (CHRC), University of Évora, 7000-801 Évora, Portugal; 6 Department of Pharmacy, Faculty of Medicine and Health Science, An-Najah National University, Nablus 00970, Palestine; faten.amer@najah.edu; 7 School of Medicine and Medical Sciences, Holy Spirit University of Kaslik, Jounieh P.O. Box 446, Lebanon; souheilhallit@usek.edu.lb; 8 Psychology Department, College of Humanities, Effat University, Jeddah 21478, Saudi Arabia; 9 Applied Science Research Center, Applied Science Private University, Amman 11937, Jordan; 10 Department of Psychiatry, College of Medicine, Qassim University, Buraidah 52571, Al Qassim, Saudi Arabia; 11 College of Education and Human Development, Princess Nourah bint Abdulrahman University, Riyadh 13415, Saudi Arabia; afaljbelh@pnu.edu.sa (A.F.A.); eaalzoaby@pnu.edu.sa (E.A.A.); 12 Department of Internal Medicine, Section Nursing Science, Erasmus University Medical Center (Erasmus MC), 3015 GD Rotterdam, The Netherlands; musheer.jaberi@gmail.com; 13 Department of Community and Public Health, Nursing College, Taibah University, Madinah 42377, Saudi Arabia; hazzminno345@gmail.com; 14 Institute of Nursing Sciences, Basic Health Sciences and Health Visiting, Faculty of Health Sciences, University of Pécs, 7622 Pécs, Hungary; annamaria.pakai@etk.pte.hu; 15 Department of Nursing, Faculty of Nursing, Jerash University, Jerash 26173, Jordan; haitham.khatatbeh@jpu.edu.jo

**Keywords:** hormonal replacement therapy (HRT), mindfulness-based stress reduction (MBSR), risk-reducing salpingo-oophorectomy (RRSO), *Breast Cancer Associated Susceptibility Protein Type 1/2* (*BRCA1/2*) mutations, menopause rating scale (MRS), menopause/perimenopause/postmenopausal, psychological symptoms, physical activity/exercise, breast cancer

## Abstract

*Breast Cancer Associated Susceptibility Proteins Type 1/2* (*BRCA1/2*) promote cellular functioning by modulating NRF2-mediated antioxidant signaling. Redox failure in women with *BRCA1/2* insufficiency increases the risk for breast/ovarian/uterine cancers. Risk-reducing salpingo-oophorectomy (RRSO) is a prophylactic surgery of the reproductive organs, which is frequently conducted by the age of 40 to lower the occurrence of cancer in women with *BRCA1/2* mutations. However, abrupt estrogen decline following RRSO causes ovarian failure, which implicates various cellular physiological processes, resulting in the increased release of free radicals and subsequent severe onset of menopausal symptoms. Comfort measures (e.g., hormonal replacement therapy (HRT) and mindfulness-based stress reduction (MBSR)) may improve chronological menopause-related quality of life, but their specific effects are not clear in women with gene mutations. Aiming to fill the gap, this study used path analysis to examine the effects of HRT and MBSR on menopausal symptoms among RRSO patients (N = 199, mean age = 50.5 ± 6.7 years). HRT directly alleviated the levels of urogenital symptoms (β = −0.195, *p* = 0.005), which mediated its indirect significant effects on the somatic–vegetative and psychological symptoms of menopause (β = −0.046, −0.067; both *p* values = 0.004, respectively), especially in BRCA2 carriers and in women who were currently physically active, premenopausal at the time of RRSO, had a high BMI, and had no history of breast cancer. It increased the severity of urogenital symptoms in women with a history of cancer. MBSR, on the other hand, was associated with indirect increases in the intensity of the somatic–vegetative and psychological symptoms of menopause (β = 0.108, 0.029; *p* = 0.003, 0.033, respectively). It exerted positive direct effects on different menopausal symptoms in multigroup analysis. The results suggest that young women undergoing recent RRSO may benefit from HRT at an individual level, while their need for extensive measures to optimize their psychological wellbeing is ongoing. The adverse effects of MBSR, which are captured in the present study, imply that MBSR may interfere with redox sensitivity associated with estradiol fluctuations in *BRCA1/2* carriers. Investigations are needed to test this hypothesis and elaborate on the underlying mechanisms in these women.

## 1. Introduction

Breast cancer is the most prevalent cancer among women [1], with one in eight US women developing invasive breast cancer over the course of their lifetime, and more than two million cases being annually diagnosed worldwide. This cancer type is the second leading cause of cancer-related female mortality [1,2,3]. *Breast Cancer Associated Susceptibility Proteins Type 1/2* (*BRCA1/2*) are closely related tumor suppressor genes. Mutations in both genes are primarily associated with hereditary breast cancers. They comprise 1863 and 3418 amino acids, in order, which interact with various cellular proteins resulting in the regulation of specific transcriptional pathways involved in cell cycle progression, highly specialized DNA repair processes, DNA damage-responsive cell cycle checkpoints, cytoplasmic division, and apoptosis, denoting their tumor-suppressing activity [4,5,6]. Compared with the general population, female carriers of BRCA1 and BRCA2 mutations exhibit an increased lifetime risk of developing breast and ovarian cancer by 59% and 16.5%, respectively [4,7,8]. *BRCA1* deficiency is associated with the activation of inflammatory signaling (tumor necrosis factor α (TNF-α) activation of nuclear factor kappa B (NF-κB) and mRNA expression of NF-κB-dependent target gene superoxide dismutase 2 (SOD2)), as well as increased production of free radicals, resulting in DNA damage, which triggers malignant transformation [9]. Carcinogenesis in *BRCA2* deficiency is associated with increased incidence of binucleated cells, alterations in chromosome number (aneuploidy), and structurally abnormal chromosomes [5]. Because of the failure of early detection by ovarian cancer screening, current guidelines recommend risk-reducing salpingo-oophorectomy (RRSO) at the age of 35–40 years for *BRCA1* mutation carriers and at 40–45 years for *BRCA2* mutation carriers as a standard approach to decrease the incidence of cancer among these women [10]. Evidence denotes that timed RRSO lowers the risk of ovarian cancer by 96% and overall mortality by 76% [8,10].

Estrogen drop following menopause accelerates ROS production, which parallels alterations in the circadian rhythm—an internal biological clock that regulates physiological processes and organ homeostasis [11,12]. Investigations involving premenopausal and postmenopausal women who do not receive hormone replacement therapy (HRT) or antioxidant supplements only identify elevated oxidative stress as a biomarker in menopausal women. Oxidative stress is associated with the severity of hot flushes and psychological stress in menopausal women [13]. Wide-scale screening of community-dwelling menopausal women in Canada uncovers the highest incidence of depression (odds ratio = 1.45; CI: 1.07–1.97) among women encountering menopause before the age of 40 [14]. Surgical menopause induced by RRSO involves sudden and sharp cessation of estrogen release, resulting in an acute onset of vasomotor, psychological, physical, and sexual symptoms, which are usually more severe than naturally occurring menopausal symptoms. Therefore, HRT is usually prescribed to mitigate the severity of menopausal symptoms [8,15].

Randomized control trials and experimental studies show that the anti-menopausal effects of HRT are linked to a mechanism that implies improved antioxidant production and decreased ROS release [16,17]. Experimental evidence reports that ovariectomy impairs the estrogen receptor (ER) expression profile by increasing the ER α/β ratio [18]. Estrogen supplementation shortly after ovariectomy, but not late supplementation, prevents the shift in the ER α/β ratio, which is associated with restoration of glutathione peroxidase and catalase activity, along with inhibition of mitochondrial hydrogen peroxide release and oxidative damage to cellular lipid and protein structures [18]. Nonetheless, few studies document benefits of short-term use of HRT (estrogen alone, not estrogen plus progesterone) on menopausal symptoms (endocrine and sexual) and on the adverse aging-related health effects of surgical menopause (e.g., on bones, cardiovascular system) among *BRCA1/2* mutation carriers, with debates on the risk of breast cancer occurrence following long-term use of HRT [19,20,21,22,23]. Noticeably, all studies, except for one longitudinal report [19], analyzed breast cancer risk following HRT in *BRCA1* carriers together with no differentiation between *BRCA1/2* and *BRCA2*. Even in a single study that included only *BRCA1* carriers, those with a history of breast cancer were not eligible to take part [19]. Therefore, history of cancer was not considered as a risk factor in the incidence of cancer post HRT treatment. Moreover, no research has examined the extent to which HRT’s beneficial effects on menopausal symptoms may be augmented/interrupted as a function of having a history of breast cancer as well as body composition and lifestyle factors.

The psychological symptoms of menopause may lower women’s general subjective wellbeing, alter their quality of life and social relations, impede their work performance, accelerate their perception and exposure to the negative effects of job demands, induce emotional exhaustion, and promote burnout [24,25,26]. Moreover, these symptoms require special attention because they may signify prodromes of age-related disorders such as Alzheimer’s disease in genetically vulnerable patients [11,15]. Interventions that ameliorate menopausal symptoms, especially related to emotional dysfunction, can improve women’s work ability [24]. Mindfulness-based stress reduction (MBSR) is a well-designed program that combines three meditation activities (body scanning exercises, sitting meditation, and gentle yoga poses). MBSR prompts the individual to pay full attention to the present moment in an accepting, non-judgmental manner [10,27]. Among euthymic women in the menopause transition, MBSR is reported to prevent the occurrence of depressive symptoms, lower perceived stress and trait anxiety, increase trait mindfulness and psychological resilience, and improve sleep, with more benefits occurring in women with sensitivity to estradiol fluctuation and excessive life stress. However, the development of major depressive episodes may not change in response to MBSR treatment [27]. Among post-menopausal women, MBSR alleviated psychological, physical, and sexual symptoms, but it had no effect on vasomotor symptoms [28]. Unlike chronological menopause, MBSR in RRSO women is reported to alleviate vasomotor and physical symptoms [10]. These symptoms are key effectors of psychological distress in the menopause period [15]. However, the effects of MBSR and HRT on menopause-related psychological symptoms are unclear in RRSO patients [10]. Given the interplay among different menopausal symptoms, the effect of vasomotor and urogenital symptoms on psychological symptoms within the context of MBSR and HRT is also unclear.

To fill this gap, the current study aims to explore the associations among the different climacteric symptoms as well as the effect of MBSR and HRT on menopausal symptoms among *BRCA1/2* carriers undergoing RRSO. Based on the available literature, we hypothesized that (1) the severity of psychological symptoms is associated with more severe physical/vegetative and urogenital symptoms, and (2) women receiving MBSR and HRT would experience less psychological and other menopausal complaints than those not administered either treatment.

## 2. Materials and Methods

### 2.1. Design, Participants, and Procedure

This cross-sectional study is a secondary analysis based on the data of 199 women with *BRCA1/2* mutations from the Netherlands who underwent RRSO and were treated in the University Medical Center Groningen (UMCG) family cancer clinic between November 2015 and December 2016. Participation was limited to those who were aged 18 to 52 years at the time of RRSO, who did not have a severe psychiatric disorder or a currently active cancer. Inadequate fluency of the Dutch language disqualified participation in the study [8].

Out of 239 women who took part in the Psychosexual Consequences of Risk-reducing Salpingo-oophorectomy (PURSUE) study, 200 returned the questionnaire, with 1 respondent excluded from the analysis because of extensive missing data. A subset of the sample comprised women with at least two moderate-to-severe menopausal symptoms (n = 23) who participated in an eight-week MBSR training program during the period between January 2015 and October 2015: the entire experimental group comprised 34 women. The intervention is described in detail elsewhere [10]. In brief, the participants attended 2.5 h weekly standard MBSR sessions, which did not specifically focus on menopausal symptoms, and a silent retreat evening of 4 h. MBSR training sessions were delivered at three locations by one of three certified and experienced MBSR trainers. Adherence to the MBSR training protocol was 80%, as ensured by audio recording 12.5% of all training sessions as well as by frequent organized meetings of the trainers under the supervision of an experienced MBSR trainer. The participants also performed daily mindfulness exercises at home for a duration of 30–45 min following instructions on an MP3 player. Participants’ adherence to the treatment regimen was monitored through recording their attendance by the trainer at the start of each session and asking them to use weekly evaluation forms to record the frequency and duration of daily home exercises—attendance and adherence to 33 min of home MBSR exercises daily were reported for 79% and 75% of the respondents [10]. The rest of the participants received care as usual (n = 176) [8]. Six participants dropped out of the treatment group because of scheduling conflicts, not having enough time for the training, and not expecting to benefit from the training, while four participants did not complete the questionnaire at the 6-month follow up for unknown reasons [10].

All patients signed an informed consent form before completing the questionnaire. Participation was voluntary, and procedures that protect patients’ privacy and confidentiality were undertaken [8]. The experimental study is registered in the ClinicalTrials.gov database with the identifier NCT02372864 [10]. The intervention and data collection protocol of the PURSUE study was approved by the Medical Ethical Committee of the UMCG on 14 November 2014 (registration no. NL46796.042.14). For the current analysis, no ethical approval was obtained because we used a public dataset shared under the terms of Creative Common License (CC BY 4.0) [29].

### 2.2. Measures

The participants completed a self-administered questionnaire, which comprised two sections. Section one inquired about various sociodemographic and clinical variables including age, weight, and height to calculate body mass index (BMI); employment; current smoking (yes/no); alcohol drinking habits (yes/no); duration of physical exercise per week; type of BRCA1/2 mutation; date of RRSO; menopausal status at the time of RRSO; history of breast cancer; and past or current use of HRT. The duration of menopausal symptoms was calculated as the time elapsed since RRSO, and data on specific breast cancer treatment (chemotherapy, hormone therapy, radiotherapy, and immunotherapy) were obtained from medical records.

Section two comprised the Menopause Rating Scale (MRS), an 11-item measure, which depicts the severity of menopausal symptoms. Items are rated on a five-point response scale (0 = no symptoms, 1 = mild, 2 = moderate, 3 = severe, and 4 = extremely severe), with total scale scores ranging between zero and 44. Higher scores signify greater symptom severity. Items are categorized into three subscales: (1) psychological symptoms (e.g., irritability, depressive mood, mental exhaustion, and anxiety), (2) somatic–vegetative/vasomotor and physical symptoms (e.g., hot flushes and sweating, heart discomfort, joint and muscle discomfort, and sleep difficulties), and (3) urogenital symptoms (e.g., vaginal dryness, sexual problems, and bladder problems).

### 2.3. Statistical Analysis

Descriptive statistics were reported as mean and standard deviation or frequency and percentage based on the type of variables. A path analysis model was conducted to examine the associations among different menopausal symptoms. Psychological symptoms were used as an outcome variable. The model also examined different effects of MBSR and HRT on menopausal symptoms. The model involved many sociodemographic and clinical variables as predictors. Some of these variables were removed from the model through a step-by-step deletion of non-significant predictors. We used multi-group analysis to examine the stability of the final model across different groups (e.g., type of mutation, history of breast cancer, menopausal status at the time of RRSO, BMI, physical activity level, and current smoking). For each model, four levels of group invariance were measured: configural, metric, scalar, and strict. Model fit was based on a non-significant Chi square (χ^2^) test, along with absolute fit indices: Comparative Fit Index (CFI), Tucker–Lewis Index (TLI), and root mean square error of approximation (RMSEA). Values indicating acceptable fit are >0.90, >0.90, and <0.08, respectively [30]. In the multigroup analysis, models with a significant χ^2^ were considered invariant if ΔCFI and ΔRMSEA were greater than 0.020 and 0.015, respectively [31]. The analysis was performed in SPSS and AMOS version 24, and significance was considered at a probability of 0.05.

## 3. Results

### 3.1. Participant Characteristics

The average age of the participants at the time of data collection and at the time of RSSO was 50.5 ± 6.7 and 42.5 ± 4.9 years, respectively. The average time elapsed since RRSO was 7.9 ± 4.8 years. Approximately half of the participants were *BRCA1* carriers (n = 102, 51.3%), one-third of the participants were *BRCA2* carriers (n = 66, 33.2%), and the mutation status was unknown in 31 women (15.6%). One-third of the participants had a history of breast cancer (n = 67, 33.3%). More than one-third of the participants reported a lifetime use of HRT (n = 81, 40.7%). Most women were currently non-smokers (n = 173, 86.9%) and premenopausal at the time of RRSO (n = 134, 67.3%). Less than two-thirds of the participants (n = 122, 61.3%) reported engagement in physical activity for more than 30 min/day for five days or more. Most of the women had a BMI of 25 or below (n = 114, 57.3%), with only three women reporting a BMI below 20, while more than one third of the participants had BMI above 25 (n = 82, 41.2%). More than two-thirds of the participants reported moderate to severe overall menopausal symptoms (n = 137, 68.9%). Severe and moderate urogenital symptoms were reported by a large portion of the sample (n = 103, 51.8% and n = 48, 24.1%, respectively). Approximately half of the participants reported moderate to severe somatic–vegetative symptoms (n = 111, 55.8%) and psychological symptoms (n = 95, 47.7%).

### 3.2. Effect of HRT and MBSR on Menopausal Symptoms (Path Analysis)

**Figure 1:**
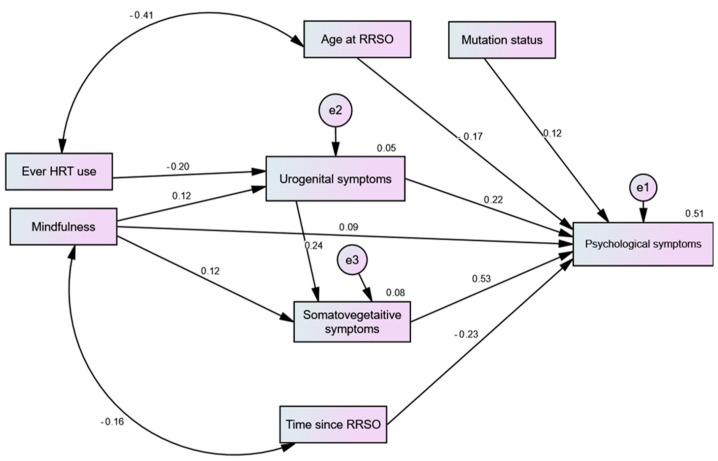
Path analysis predicting the effects of mindfulness-based stress reduction (MBSR) and hormonal replacement therapy (HRT) on different menopausal symptoms among women with risk-reducing salpingo-oophorectomy (RRSO).

After trimming non-significant sociodemographic predictors, the path model had an excellent fit (CFI = 1.00, TLI = 1.00, RMSEA = 0.00, χ^2^ = 14.18, *p* = 0.585). As shown in Figure 1, urogenital and somatic–vegetative symptoms had significant direct effects on psychological symptoms. The direct effects of MBSR on all of the symptoms were non-significant (*p*-value range = 0.071 to 0.094). However, it had positive significant indirect effects on the psychological and somatic–vegetative symptoms (β = 0.108, *p* = 0.003 and β = 0.029; *p* = 0.033, respectively). HRT only had a significant negative direct effect on urogenital symptoms (*p* = 0.005), which mediated the negative indirect effects of HRT on psychological and somatic–vegetative symptoms (β = −0.067, *p* = 0.004 and β = −0.046; *p* = 0.004, respectively). Both age at RRSO and time elapsed after RRSO negatively predicted psychological symptoms (both *p*-values = 0.001), but they had no effect on other menopausal symptoms (paths trimmed). The same was true for mutation type (*p* = 0.018), albeit its effect was positive.

### 3.3. Effect of HRT and MBSR on Menopausal Symptoms across Groups (Multigroup Analysis)

Supplementary Table S1 indicates adequate fit of the path model across various groups, at least at the configural and metric levels. In the subgroup analysis, MBSR directly increased somatic–vegetative symptoms in *BRCA1* mutation carriers. Among *BRCA2* carriers, MBSR caused direct and indirect increases in urogenital and psychological symptoms, respectively. HRT caused significant reductions in urogenital and psychological symptoms only in *BRCA2* mutation carriers (Table 1). Among women with a history of breast cancer, MBSR predicted a direct increase in somatic–vegetative symptoms along with direct and indirect increases in psychological symptoms. Psychological symptoms were the only menopausal symptom increased as a result (indirect) of MBSR in non-cancer women. HRT resulted in a significant decline in all menopausal symptoms among women with no history of breast cancer, while it significantly increased urogenital symptoms in those with a history of breast cancer (Table 2).

MBSR increased the intensity of all menopausal symptoms in women who were menopausal and premenopausal at the time of RRSO, albeit the urogenital symptoms were not significantly affected in the latter. The effects of MBSR on psychological symptoms in both groups were only indirect. HRT resulted in a significant decline in all menopausal symptoms among women who were premenopausal at the time of RSSO (Table 3).

Among normal-weight women, MBSR was associated with a direct increase in urogenital symptoms and indirect increases in somatic–vegetative and psychological symptoms, respectively. Meanwhile, it was associated with a direct increase in somatic–vegetative symptoms in overweight women. On the other hand, HRT was associated with a direct decrease in urogenital symptoms as well as with indirect decreases in somatic–vegetative and psychological symptoms in the overweight/obese group (Table 4).

MBSR was associated with an indirect increase in the intensity of psychological symptoms in women with both low and high levels of physical activity: psychological symptoms were directly elevated in physically inactive women. HRT was associated with direct and indirect reductions in urogenital and psychological symptoms, in order, among women with low levels of physical activity only (Table 5).

Among women who smoked, MBSR caused a direct decrease in somatic–vegetative symptoms and a direct increase in urogenital symptoms, which was associated with an indirect increase in somatic–vegetative symptoms—i.e., the flare of urogenital symptom mediated the indirect effect of MBSR on somatic–vegetative symptoms. On the other hand, MBSR directly increased somatic–vegetative symptoms and indirectly increased psychological symptoms in the non-smoking group. HRT was associated with considerable reductions in all menopausal symptoms only in non-smoking women (Table 6).

Table 7 reports the standardized effects of age at RRSO and time since RRSO on psychological menopausal symptoms across different groups. Age at RRSO and time after RRSO negatively predicted psychological symptoms in BRCA1 carriers, women without a history of cancer, those who were premenopausal at the time of RRSO, current non-smokers, and those who exercised for more than 4 days. Normal-weight women who were older at RRSO had significantly fewer psychological symptoms than their counterparts with higher BMI, regardless of the time elapsed since RSSO.

## 4. Discussion

This research aimed to examine the interaction among climacteric symptoms as well as the impact of HRT and MBSR on menopausal symptoms in women with *BRCA1*/*2* gene mutations who had undergone RRSO. Psychological climacteric symptoms appeared as a direct and indirect effect of the urogenital and somatic–vegetative menopausal symptoms, which were all expressed in moderate-to-severe intensity, especially when women were young and a short time had elapsed after RRSO. HRT reduced the intensity of all types of menopausal symptoms in general, but it exacerbated urogenital symptoms in breast cancer survivors. Unexpectedly, MBSR aggravated menopausal symptoms in both *BRCA1/2* mutations, especially in breast cancer survivors. The negative effects of MBSR were consistent regardless of women’s level of physical activity, body weight, current smoking status, and menopausal status at the time of RSSO.

Our results highlight the significant positive effects of HRT on all menopausal symptoms after RRSO, which were more profound among *BRCA2* carriers, women who were premenopausal at the time of RRSO, or were physically active, whereas smoking was associated with diminished benefits of HRT. These findings are consistent with reports of former studies on HRT benefits for climacteric symptoms in chronological menopause [8,32] and in *BRCA1/2* carriers after RRSO [19,20,21,22,23]. The time elapsed since RRSO and age at RRSO consistently predicted psychological symptoms in women with *BRCA1*, those with no history of breast cancer, and those who were premenopausal at RRSO. Thus, greater benefits of HRT in RRSO patients, especially those who were premenopausal, may be achieved through early HRT administration along with a healthy lifestyle. This is because the mechanism involved in HRT benefits probably entails maintaining/improving overall physiological functioning. Experimentally, early HRT treatment in ovariectomized rats inhibits the shift in the estrogen receptor (ER) α/β ratio and mitochondrial-related oxidative destruction of the cellular lipid and protein structures [18], which is associated with improvements in voluntary physical activity, uterine growth, and metabolism (protein expression responsible for the browning of white fat and insulin signaling, including the hepatic expression of fibroblast growth factor 21) [33].

The negative effects of HRT that appeared in those with a history of breast cancer and in *BRCA1* carriers in our study may be attributed to excessive baseline levels of inflammation and oxidative stress associated with cancer treatment [34] and *BRCA1* deficiency—*BRCA1* protects against neuronal damage through the activation of the Antioxidant Response Element signaling pathway, as it interacts with the nuclear factor (erythroid-derived 2)-like 2 (NRF2) through the *BRCA1* C-terminal (BRCT) domain [6]. Estrogen, as a neuroendocrine molecule, affects neuronal metabolism and functioning [11]. Therefore, estrogen drop following RRSO in *BRCA1* carriers may be associated with more severe (psychological) menopausal symptoms secondary to excessive neuronal alterations, especially within the context of intense redox failure associated with the absence of the antioxidant effects of *BRCA1* [6]. This justification seems feasible, since HRT aggravated urogenital symptoms in participants with a history of breast cancer, whereas HRT is usually indicated for severe menopausal symptoms. In this sense, our results indirectly signify that HRT may potentially increase the already elevated risk of breast cancer in BRCA1 carriers who have a history of cancer, even when estrogen alone may be safe relative to estrogen plus progesterone (12% vs. 22% 10-year incidence of breast cancer) in *BRCA1* carriers who never had cancer. This notion obtains support from a meta-analysis of four RCTs with 4050 breast cancer survivors, which associates HRT with cancer recurrence in patients with hormone-receptor-positive tumors but not in those with hormone-receptor-negative disease [35]. Supplementary Material S2 thoroughly elaborates on the mechanisms underlying severe menopausal symptoms following HRT treatment in *BRCA1* and in carcinogenesis.

Psychosocial interventions addressing menopausal symptoms and mental wellbeing after RRSO in *BRCA1/2* carriers are scarce, with mixed results ranging from no to minimal effects for most outcomes [36,37]. MBSR is known as an effective meditation intervention [38]. Despite its reported beneficial effects on psychological and physical symptoms among breast cancer survivors [39], our results indicate that MBSR may not be beneficial or may even be harmful after RRSO in *BRCA1/2* carriers. In support of our findings, the effect of MBSR on the symptoms of depression, stress, fatigue, and cognitive impairment in breast cancer survivors is moderated by three genetic polymorphisms [40]. Likewise, a lack of response to MBSR is documented in a considerable proportion of veterans with post-traumatic stress disorder [38], with genetic evidence associating MBSR with stress-related pathways at the molecular level [38,40]. Non-response to MBSR in veterans is related to increased methylation in *FKBP5* intron 7 bin 2 at CpG_35558513 site (containing known glucocorticoid response elements, GREs) [38]. 

*BRCA1* enhances glucocorticoid receptor (GR) levels and GR transcriptional activity, which suppresses the levels of pro-inflammatory cytokines [41] and regulates circadian rhythm, stress response, and organ homeostasis [42]. On the other hand, *BRCA1* deficiency is associated with GR dysregulation, which accelerates inflammatory and oxidative signaling during the luteal phase, resulting in prolonged postovulatory inflammation in the nonmalignant fallopian tube epithelium [9,41]. GR dysregulation in *BRCA1* mutation carriers with breast cancer reduces the phosphorylation of GR at the Ser-211 position compared with women without breast cancer [42]. Meanwhile, stress perceptions are persistently high in a considerable proportion of *BRCA1/2* cases apart from exposure to *BRCA1/2* genetic test results [43,44], which may justify the increased prevalence of problems associated with memory and concentration following RRSO [45], particularly as executive dysfunction in these patients is mediated by mood dysregulations pertinent to early life stress [46]. Chronic psychosocial stress persistently activates GR signaling, allowing glucocorticoid dysregulation to trigger negative physiological and pathological effects, including the development of aggressive and drug-resistant cancers [47,48]. Taken together, poor response to MBSR in *BRCA1/2* carriers probably implicates an insult of the hypothalamic–pituitary–adrenal (HPA) axis, which is associated with GR dysregulation secondary to the baseline stress propensity of *BRCA1/2* mutations, which may be further exacerbated by an interplay with MBSR-induced methylation.

### Strengths, Implications, and Limitations

This study has the merit of employing already available public data to investigate the effects of HRT and MBSR on menopausal symptoms among *BRCA1/2* carriers who underwent RRSO. The results revealed that MBSR was ineffective/harmful, while HRT exerted positive effects in certain groups. The findings emphasize the significance of the early initiation of HRT and a healthy lifestyle to maximize its benefits for the alleviation of climacteric symptom in RRSO patients, particularly young women who are premenopausal at the time of surgery. Lifestyle (e.g., physical activity and smoking) was an important factor that interfered with the effects of HRT. Although exercise activates the signaling cascades involved in the correction of metabolic dysfunctions, autophagy, and the production of antioxidants—which all promote optimal physiological functioning [49,50,51]—none of the menopausal symptoms correlated with engagement in physical activity for 30 min or more per day for 5 days or more, which was reported by 61.1% of participants. Thus, participating in physical activity alone does not seem to be effective for alleviating menopausal symptoms in this population. Nonetheless, it is worth mentioning that categorizing physical activity into two groups (0–4 days; 5 days or more) in the current study might preclude estimating the differences between those who do not participate in physical activity at all and those who do. Additionally, the level of physical activity (mild, moderate, and strenuous) was not evaluated, which may be considered in future studies.

Because of the likely negative effects of HRT in women with a history of cancer, there is an ongoing need to search for possible alternatives to HRT that are worthy of investigation for their anti-menopausal effects in this population, such as natural phytochemicals/phytoestrogens, a large family of molecules that exist in natural products (e.g., royal jelly, bee honey, soybeans, floral pollen) that mimic the physiological effects of estrogen [11,52]. Natural agents with potent anti-inflammatory activities (e.g., propolis, black cumin seeds, ginger,) may also be particularly helpful [53]. This is because *BRCA1* mutations may fulminate climacteric symptoms in RRSO patients secondary to the activation of inflammatory signaling in the fallopian tube epithelium [9]. Given the dearth of effective interventions for distress, fatigue, sleep difficulties, depression, and anxiety in this population [36,37], those suffering excessive psychological distress may need specialized counseling. Traditional alternative modalities such as foot herbal water baths, foot massage/reflexology, and acupuncture, which are reported to exert anti-menopausal effects [52], represent other options that deserve research attention to alleviate the suffering associated with sharp menopausal onset following RRSO among women with *BRCA1/2* gene mutations. To successfully convey intricate genetic and cancer risk information related to *BRCA1/2* mutation, it is imperative that we gain a deeper understanding of the distinct requirements of this group and locate helpful tools to assist them in navigating the intricate clinical realm concerning risk reduction and symptom management.

This study has some limitations, which may call for caution when interpreting the findings. Although an experimental design was originally initiated, we could only use post-experimental cross-sectional data. The lack of pre-treatment measures of menopausal symptoms may confound the reports. The data of 11 participants from the original experimental group were missing, so attrition risk may also bias the results. Data collection took place over a long period of time following the intervention. Thus, the results may be influenced by the time–treatment effect. Some variables were vaguely defined, e.g., physical activity was assessed as 30 min per day regardless of the level of the activity. Data on compliance with MBSR show minor violations of the training protocol and minor lack of adherence by the subjects, while details of women’s satisfaction with this therapy were entirely missing. In addition, the study uses data from a specific demographic, which compromises the generalizability of results to broader populations since cultural traditions largely influence individuals’ experiences and perceptions (e.g., of disease, treatment, and own fate) [54]. Lastly, the long-term effects of MBSR and HRT on menopausal discomfort need to be carefully assessed, particularly as the effects of comfort measures in this population show absurd fluctuations, e.g., the absence of effect immediately and 12-months post treatment and the occurrence of improvement six months after the intervention. Making use of decision aids that utilize tailored information on hereditary risk may facilitate the accurate monitoring of treatment effects [36,37].

## 5. Conclusions

The results uncover the high prevalence of menopausal symptoms following RRSO in *BRCA1/2* mutation carriers, with psychological distress frequently mirroring the severity of somatic–vegetative and urogenital discomfort. Menopausal symptoms following RRSO should not be underestimated given their significant influence on women’s quality of life. This realization emphasizes the need for individualized interventions that target the various facets of menopausal symptoms. HRT arises as a ray of hope for alleviating menopausal symptoms in specific subgroups: *BRCA2* carriers, premenopausal, physically active, and nonsmoking women. Accordingly, lifestyle factors such as physical activity and smoking have a substantial impact on its efficacy. Moreover, the administration of HRT necessitates a nuanced evaluation of its potential benefits and risks, particularly for those with a breast cancer history, highlighting the significance of individualized care in *BRCA1/2* carriers. On the other hand, MBSR presents a more complex picture, implying that its utility may not be ubiquitous, and that caution should be exercised when contemplating it as an intervention for this specific population. Our study emphasizes ongoing efforts to improve the wellbeing of *BRCA1/2* carriers, highlighting the critical need for further investigations in refining the mechanisms underlying therapeutic strategies and providing individualized solutions to alleviate suffering.

## Figures and Tables

**Table 1:** Multigroup analysis examining the effects of mindfulness-based stress reduction (MBSR) and hormone replacement therapy (HRT) across groups of *BRCA1/2* mutation carriers.

Predictor	Type of Mutation	Type of Effect	Outcome Variables								
			Urogenital			Somatic–Vegetative			Psychological		
			β	*p*	95% CI	β	*p*	95% CI	β	*p*	95% CI
MBSR	*BRCA1*	Direct	−0.006	0.905	−0.200 to 0.160	0.164	0.039	0.010 to 0.330	0.089	0.263	−0.065 to 0.230
		Indirect				−0.001	0.892	−0.058 to 0.045	0.085	0.071	−0.006 to 0.173
	*BRCA2*	Direct	0.304	**0.001**	0.157 to 0.439	0.112	0.259	−0.073 to 0.311	0.116	0.158	−0.039 to 0.285
		Indirect				0.066	0.052	−0.001 to 0.172	0.172	**0.010**	0.042 to 0.305
HRT	*BRCA1*	Direct	−0.160	0.119	−0.342 to 0.031						
		Indirect				−0.039	0.078	−0.124 to 0.003	−0.049	0.089	−0.133 to 0.005
	*BRCA2*	Direct	−0.291	**0.019**	0.498 to −0.049						
		Indirect				−0.063	0.063	−0.200 to 0.003	−0.102	**0.018**	−0.257 to −0.011

MBSR: Mindfulness-based stress reduction; HRT: hormone replacement therapy; bold-face values reflect significant results.

**Table 2:** Multigroup analysis examining the effects of mindfulness-based stress reduction (MBSR) and hormone replacement therapy (HRT) according to history of breast cancer.

Predictor	Breast Cancer History	Type of Effect	Outcome Variables								
			Urogenital			Somatic–Vegetative			Psychological		
			β	*p*	95% CI	β	*p*	95% CI	β	*p*	95% CI
MBSR	Yes	Direct	0.105	0.191	−0.073 to 0.227	0.211	**0.004**	0.054 to 0.362	0.163	**0.045**	0.003 to 0.311
		Indirect				−0.004	0.565	−0.049 to 0.020	0.113	**0.004**	0.038 to 0.216
	No	Direct	0.156	0.082	−0.018 to 0.283	0.086	0.175	−0.038 to 0.222	0.054	0.351	−0.063 to 0.171
		Indirect				0.049	0.054	−0.001 to 0.117	0.108	**0.012**	0.019 to 0.189
HRT	Yes	Direct	0.169	**0.024**	0.026 to 0.277						
		Indirect				−0.006	0.701	−0.052 to 0.034	0.008	0.634	−0.039 to 0.060
	No	Direct	−0.191	**0.030**	−0.351 to −0.022						
		Indirect				−0.060	**0.019**	−0.133 to −0.010	−0.078	**0.022**	−0.158 to −0.012

MBSR: Mindfulness-based stress reduction; HRT: hormone replacement therapy; bold-face values reflect significant results.

**Table 3:** Multigroup analysis examining the effects of mindfulness-based stress reduction (MBSR) and hormone replacement therapy (HRT) according to menopausal status at the time of RRSO.

Predictor	Menopausal Status	Type of Effect	Outcome Variables								
			Urogenital			Somatic–Vegetative			Psychological		
			β	*p*	95% CI	β	*p*	95% CI	β	*p*	95% CI
MBSR	Menopausal	Direct	0.201	**0.038**	0.009 to 0.375	0.234	**0.017**	0.033 to 0.470	0.059	0.626	−0.179 to 0.306
		Indirect				0.045	0.080	−0.004 to 0.156	0.202	**0.006**	0.046 to 0.383
	Premenopausal	Direct	0.134	0.124	−0.032 to 0.263	0.141	**0.031**	0.011 to 0.276	0.111	0.099	−0.018 to 0.229
		Indirect				0.032	0.082	−0.003 to 0.091	0.120	**0.002**	0.041 to 0.204
HRT	Menopausal	Direct	−0.081	0.575	−0.461 to 0.199						
		Indirect				−0.018	0.396	−0.170 to 0.036	−0.037	0.472	−0.270 to 0.080
	Premenopausal	Direct	−0.212	**0.016**	−0.366 to −0.032						
		Indirect				−0.051	**0.016**	−0.123 to −0.007	−0.069	**0.010**	−0.147 to −0.012

MBSR: Mindfulness-based stress reduction; HRT: hormone replacement therapy; bold-face values reflect significant results.

**Table 4:** Multigroup analysis examining the effects of mindfulness-based stress reduction (MBSR) and hormone replacement therapy (HRT) according to body mass index.

Predictor	Body Mass Index	Type of Effect	Outcome Variables								
			Urogenital			Somatic–Vegetative			Psychological		
			β	*p*	95% CI	β	*p*	95% CI	β	*p*	95% CI
MBSR	≤25	Direct	0.203	**0.001**	0.104 to 0.303	0.075	0.217	−0.044 to 0.212	0.140	0.084	−0.021 to 0.285
		Indirect				0.042	0.031	0.004 to 0.098	0.098	**0.006**	0.024 to 0.180
	>25	Direct	−0.008	0.919	−0.213 to 0.164	0.179	**0.020**	0.023 to 0.339	0.039	0.510	−0.084 to 0.152
		Indirect				−0.002	0.920	−0.063 to 0.054	0.099	0.073	−0.009 to 0.205
HRT	≤25	Direct	−0.068	0.489	−0.540 to −0.125						
		Indirect				−0.014	0.355	−0.083 to 0.013	−0.020	0.415	−0.094 to 0.030
	>25	Direct	−0.351	**0.003**	−0.366 to −0.032						
		Indirect				−0.104	**0.003**	−0.217 to −0.028	−0.130	**0.001**	−0.258 to −0.041

MBSR: Mindfulness-based stress reduction; HRT: hormone replacement therapy; bold-face values reflect significant results.

**Table 5:** Multigroup analysis examining the effects of mindfulness-based stress reduction (MBSR) and hormone replacement therapy (HRT) according to physical activity level.

Predictor	Physical Activity Level	Type of Effect	Outcome Variables								
			Urogenital			Somatic–Vegetative			Psychological		
			β	*p*	95% CI	β	*p*	95% CI	β	*p*	95% CI
MBSR	Yes	Direct	0.134	0.145	−0.058 to 0.275	0.132	0.076	−0.015 to 0.291	0.052	0.548	−0.113 to 0.211
		Indirect				0.046	0.080	−0.007 to 0.125	0.120	**0.042**	0.004 to 0.230
	No	Direct	0.090	0.301	−0.077 to 0.233	0.129	0.061	−0.009 to 0.274	0.112	**0.043**	0.003 to 0.221
		Indirect				0.016	0.198	−0.008 to 0.065	0.099	**0.006**	0.022 to 0.182
HRT	Yes	Direct	−0.162	0.205	−0.390 to 0.082						
		Indirect				−0.056	0.129	−0.171 to 0.018	−0.070	0.176	−0.199 to 0.029
	No	Direct	−0.201	**0.019**	−0.363 to −0.022						
		Indirect				−0.035	0.061	−0.102 to 0.001	−0.059	**0.012**	−0.137 to −0.008

MBSR: Mindfulness-based stress reduction; HRT: hormone replacement therapy; bold-face values reflect significant results.

**Table 6:** Multigroup analysis examining the effects of mindfulness-based stress reduction (MBSR) and hormone replacement therapy (HRT) according to smoking status.

Predictor	Smoking	Type of Effect	Outcome Variables								
			Urogenital			Somatic–Vegetative			Psychological		
			β	*p*	95% CI	β	*p*	95% CI	β	*p*	95% CI
MBSR	Yes	Direct	0.292	**0.002**	0.099 to 0.554	−0.289	**0.029**	−0.526 to −0.013	0.097	0.462	−0.182 to 0.438
		Indirect				0.202	**0.002**	0.057 to 0.479	0.008	0.983	−0.282 to 0.264
	No	Direct	0.086	0.193	−0.050 to 0.189	0.161	**0.001**	0.055 to 0.272	0.098	0.074	−0.009 to 0.200
		Indirect				0.018	0.108	−0.004 to 0.055	0.114	**0.001**	0.049 to 0.182
HRT	Yes	Direct	0.035	0.876	−0.403 to 0.502						
		Indirect				0.024	0.857	−0.308 to 0.322	0.019	0.829	−0.246 to 0.256
	No	Direct	−0.247	**0.004**	−0.381 to −0.067						
		Indirect				−0.051	**0.006**	−0.111 to −0.011	−0.084	**0.002**	−0.152 to −0.030

MBSR: Mindfulness-based stress reduction; HRT: hormone replacement therapy; bold-face values reflect significant results.

**Table 7:** Standardized total effects of age at RRSO and time elapsed since RRSO on psychological menopausal symptoms across different groups among women undergoing RRSO.

Predictor	Group	β	*p*	95% CI
	**Type of mutation**			
Age at RRSO	BRCA1	−0.224	**0.002**	−0.393 to −0.077
	BRCA2	−0.122	0.234	−0.351 to 0.083
Time since RRSO	BRCA1	−0.210	**0.003**	−0.342 to −0.067
	BRCA2	−0.084	0.316	−0.258 to 0.081
	**Menopausal status**			
Age at RRSO	Yes	−0.010	0.937	−0.307 to 0.251
	No	0.064	**0.001**	−0.357 to −0.151
Time since RRSO	Yes	−0.176	0.215	−0.458 to 0.087
	No	0.053	**0.001**	−0.379 to −0.135
	**Body mass index**			
Age at RRSO	≤25	−0.189	**0.006**	−0.331 to −0.052
	>25	−0.142	0.120	−0.307 to 0.034
Time since RRSO	≤25	−0.154	**0.031**	−0.278 to −0.014
	>25	−0.309	**0.001**	−0.438 to −0.171
	**Cancer history**			
Age at RRSO	Yes	−0.065	0.475	−0.245 to 0.109
	No	−0.234	**0.001**	−0.368 to −0.104
Time since RRSO	Yes	−0.275	**0.003**	−0.454 to −0.097
	No	−0.239	**0.001**	−0.346 to −0.124
	Current smoking			
Age at RRSO	Yes	−0.243	0.327	−0.518 to 0.307
	No	−0.156	**0.011**	−0.271 to −0.036
Time since RRSO	Yes	−0.133	0.507	−0.451 to 0.266
	No	−0.221	**0.001**	−0.320 to −0.117
	Physical activity			
Age at RRSO	0 to 4 days	−0.095	0.256	−0.266 to 0.072
	>4 days	−0.185	**0.005**	−0.316 to −0.055
Time since RRSO	0 to 4 days	−0.311	**0.001**	−0.452 to −0.166
	>4 days	−0.162	**0.013**	−0.278 to −0.041

RRSO: Risk-reducing salpingo-oophorectomy; Breast Cancer Associated Susceptibility Proteins Type 1/2 (BRCA1/2); bold-face values reflect significant results

## Data Availability

The dataset supporting the conclusions of this article is available in Mendeley repository [29] [https://data.mendeley.com/datasets/6wtd46ry6s] (accessed on 27 June 2024), and the datasets used and/or analyzed during the current study are available from the corresponding author on reasonable request.

## Supplementary Materials

The following supporting information can be downloaded at https://www.mdpi.com/article/10.3390/healthcare12161612/s1: Supplementary Table S1: Fit indices of multi-group analysis models examining the differences in the effects of mindfulness-based stress reduction (MBSR) and hormone replacement therapy (HRT) among women with RRSO, File S2: Possible mechanism of menopausal symptom flaring following HRT treatment in RRSO patients with *BRCA1* mutation and a history of cancer.
