# Supplementary material for: Effects of Hormonal Replacement Therapy and Mindfulness-Based Stress Reduction on Climacteric Symptoms Following Risk-Reducing Salpingo-Oophorectomy
Source: Healthcare (Basel). 2024 Aug 13;12(16):1612. doi: 10.3390/healthcare12161612 (PMC11353799; doi:10.3390/healthcare12161612)
Supplement: Supplementary file 1 [file healthcare-12-01612-s001.zip › healthcare-3103893-supplementary.pdf]

Supplementary File S1.

Table S1. Fit indices of multi-group analysis models examining the differences in the effects of mindfulness-based stress reduction (MBSR) and hormone replacement therapy (HRT) across different among women with RRSO.

Groups	Invariance levels	χ^2	df	p	$\Delta\chi^2$	Δdf	$p(\Delta\chi^2)$	CFI	ΔCFI	TLI	ΔTLI	RMSEA	ΔRMSEA
Type of mutation	Configural	27.930	20	0.111	-	-	-	0.951	-	0.897	-	0.049	-
	Metric	33.806	29	0.246	5.875	9	0.752	0.970	-0.019	0.957	0.040	0.032	0.017
	Scalar	48.004	35	0.070	14.199	6	0.027	0.920	0.050	0.904	0.053	0.047	-0.015
	Strict	50.637	38	0.082	2.633	3	0.452	0.922	-0.002	0.914	-0.010	0.045	0.002
Breast cancer history	Configural	29.170	32	0.610	-	-	-	1.000	-	1.025	-	0.000	-
	Metric	47.536	42	0.257	18.366	10	0.049	0.972	0.002	0.962	0.063	0.026	-0.026
	Scalar	69.126	49	0.031	21.589	7	0.003	0.897	0.005	0.882	0.000	0.046	-0.020
	Strict	73.657	52	0.026	4.531	3	0.210	0.889	0.031	0.880	0.026	0.046	0.000
Menopausal status	Configural	30.581	32	0.538	-	-	-	1.000	-	1.015	-	0.000	-
	Metric	37.482	42	0.669	44.727	16	0.001	1.000	0.000	1.036	-0.021	0.000	0.000
	Scalar	52.963	49	0.324	67.076	21	0.001	0.976	0.024	0.973	0.063	0.022	-0.022
	Strict	56.165	52	0.322	332.818	27	0.001	0.975	0.001	0.973	0.000	0.022	0.000
Body mass index	Configural	28.101	32	0.664	-	-	-	1.000	-	1.000	-	0.000	-
	Metric	39.939	42	0.562	11.837	10	0.296	1.000	0.000	1.000	0.000	0.000	0.000
	Scalar	45.125	49	0.631	5.186	7	0.637	1.000	0.000	1.000	0.000	0.000	0.000
	Strict	46.141	52	0.703	1.016	3	0.797	1.000	0.000	1.000	0.000	0.000	0.000
Physical activity	Configural	29.873	32	0.575	-	-	-	1.000	-	1.020	-	0.000	-
	Metric	34.655	42	0.782	4.782	10	0.905	1.000	0.000	1.054	0.034	0.000	0.000
	Scalar	44.088	49	0.672	9.433	7	0.223	1.000	0.000	1.031	0.023	0.000	0.000
	Strict	48.314	52	0.620	4.226	3	0.238	1.000	0.000	1.022	0.011	0.000	0.000
Smoking	Configural	34.851	32	0.334	-	-	-	0.986	-	0.975	-	0.021	-
	Metric	47.667	42	0.253	12.816	10	0.234	0.972	0.014	0.963	0.012	0.026	-0.005
	Scalar	52.251	49	0.349	4.584	7	0.711	0.984	-0.012	0.982	-0.021	0.018	0.008
	Strict	57.082	52	0.292	4.831	3	0.185	0.975	0.009	0.973	0.011	0.022	0.004

Supplementary File S2.

On one hand, severe menopausal symptoms may be linked to estrogen metabolism enzyme polymorphisms, which are associated with an excessive overproduction of reactive oxygen species (ROS) and inappropriate detoxification due to the reduced production of internal antioxidant enzymes [13,40,41]. As an example, the improper inactivation of catechol estrogen quinones (which are specific reactive estrogen metabolites) causes a surge in ROS, which interact with DNA and promote the initiation of cancer by inducing mutations in critical genes [41]. From another perspective, estrogen and progesterone receptor (ER, PR) signaling regulates mammary cell development and interferes with breast carcinogenesis [3,42]. It regulates different mechanisms regarding the generation of ROS and corrosive free radicals, which promote carcinogenesis [40,41]. Accordingly, ER-targeted endocrine therapies have been employed to significantly improve clinical outcomes in breast cancer [3]. However, HRT may increase cancer risk in menopausal women [17,43]. Concerning the target population of our investigation, a diagnostic meta-analysis of prospective studies reports an increased incidence of endometrial carcinoma in BRCA carriers receiving certain HRT types (e.g., tamoxifen) [44]. Likewise, a meta-analysis involving four RCTs with 4,050 breast cancer survivors associates HRT with cancer recurrence in patients with hormone receptor-positive tumors (HR 1.8, 95% CI 1.15–2.82, $p = 0.010$) but not in those with hormone receptor-negative disease (HR 1.19, 95% CI 0.80–1.77, $p = 0.390$) [29]. Accordingly, a pre-clinical trial involving the administration of physiologically relevant levels of E2 and progesterone (P4) to immunocompromised mice, which were formerly injected with ER plus breast cancer cell lines and patient-derived tumor cells in milk ducts, resulted in increased tumor growth and metastatic spread. The proliferative mechanism was patient-specific, with low MYC family oncogene and androgen receptor signatures promoting transcription and proliferation upon stimulation with P4 [42].

HRT seems to aggravate oxidative stress in BRCA1/2 women with cancer who already express higher baseline levels of inflammation and oxidative stress, resulting in the potentiation of menopausal symptoms. In support of this logic, HRT significantly alleviated menopausal symptoms in participants without a history of breast cancer. Indeed, a large-scale study comprising 616 patients with confirmed breast cancer and 1,082 age- and race-matched normal controls revealed that lifetime use and 5-year use of HRT are associated with an increased risk of breast cancer. Alternatively, polymorphisms in the promoter region of the CAT gene (rs1001179) were not associated with breast cancer. However, CAT genotyping revealed a significant increase in the incidence of estrogen receptor-positive tumors in women with CT or TT variants who received HRT compared with women with a CC allele who received HRT, suggesting that HRT's contribution to breast cancer involves the dysfunctional modulation of oxidative stress [45]. Therefore, HRT use among BRACA1/2 carriers may be considered at an individual level.
